# Shelf-Life Evolution of the Fatty Acid Fingerprint in High-Quality Hazelnuts (*Corylus avellana* L.) Harvested in Different Geographical Regions

**DOI:** 10.3390/foods10030685

**Published:** 2021-03-23

**Authors:** Marta Cialiè Rosso, Federico Stilo, Steven Mascrez, Carlo Bicchi, Giorgia Purcaro, Chiara Cordero

**Affiliations:** 1 Dipartimento di Scienza e Tecnologia del Farmaco, Università degli Studi di Torino, Via Pietro Giuria 9, I-10125 Torino, Italy; marta.cialierosso@unito.it (M.C.R.); federico.stilo@unito.it (F.S.); carlo.bicchi@unito.it (C.B.); 2 AgroBioChem Department, Gembloux Agro-Bio Tech, University of Liège, Passage des Déportés 2, 5030 Gembloux, Belgium; steven.mascrez@uliege.be

**Keywords:** free fatty acids, hazelnut lipids, esterified fatty acids, fatty acids methyl esters, quantitative profiling, hazelnut drying, storage quality of hazelnuts

## Abstract

Hazelnuts are characterized by a relatively high abundance of oleic acid and poly-unsaturated fatty acids, which give this fruit a high nutritional value. As a counterbalance, such a lipid profile is more susceptible to autoxidation and/or degradation reactions under enzymatic catalysis. Lipid oxidation occurs on fatty acids (FAs), both esterified on triacylglycerols and in free form (after lipolysis), but with favorable kinetics on the latter. In this study, the quali-quantitative changes in FA profiles (both free and esterified) were monitored during the shelf life (time 0, 6, and 12 months) as a function of different drying and storage conditions and different cultivars and geographical areas. A derivatization/extraction procedure was performed to quantify the profile of free and esterified fatty acids accurately. The overall profile of the free and esterified fatty acids concurred to create a biological signature characteristic of the cultivar and of the harvest region. The free and esterified forms’ characterization enabled the efficient monitoring of the effects of both the hydrolytic activity (increment in overall free fatty acids) and the oxidative process (decrease in unsaturated free fatty acids versus esterified fatty acids) over the 12 months of storage.

## 1. Introduction

Hazelnuts (*Corylus avellana* L.) are characterized by high fat content, with triacylglycerols as the main components [1]. Moreover, the relatively high abundance of mono- and unsaturated fatty acids gives this fruit a high nutritional value, as well as a great susceptibility to autoxidation and/or degradation reactions under enzymatic catalysis. The formation of secondary lipid oxidation products, mainly carbonyl derivatives [2], can affect hazelnut sensory quality, representing a challenging issue for confectionary industries [3]. Many studies have been carried out to characterize the lipid fraction of hazelnut and to evaluate its stability during the shelf life [4,5,6].

State-of-the-art literature unequivocally indicates the geographical origin and environmental conditions as the most influencing variables on the lipid composition [7,8] in terms of acidic profile. At the same time, storage, water activity (*a_w_*), and temperature [4,6] have a clear and decisive impact on its chemical stability [9].

Free fatty acids (FFAs), peroxide value, and esterified fatty acid (EFA) profiling through fatty acid methyl ester (FAME) derivatives, are the most common indicators in the quality monitoring of fats. FFAs are generally recognized as useful indicators of quality loss in edible oils and fats, as lipid oxidation is more extensive in free acids, resulting in undesirable taste and aroma. Özdemir et al. [10,11], investigating hazelnut lipid stability and quality, considered a free titratable acidity ≥ 1% as an indicator of rancidity. Turan [5] observed an increase in free acidity percentage from 0.04 to 0.36% during 24-month storage of hazelnuts from the Ordu region (Turkey) when submitted to post-harvest industrial drying under controlled kinetics of dehydration and final moisture values.

Lipid rancidity in natural products is a combination of the activity of two main enzymes (beyond other external factors), namely lipase and lipoxygenase, which lead to hydrolytic rancidity and oxidation, respectively [2,12]. Radical oxidation may occur on triacylglycerols (TAGs) or FFAs, but it has been shown that the oxidation kinetics is favorable on FFAs, which act as pro-oxidants [12,13]. However, if researchers are interested in not only the simple hydrolytic activity but also its impact on the glycerolipid fraction, these parameters are not sufficiently accurate to provide information about the quali-quantitative changes in fatty acid (FA) profile/composition. An attempt to go beyond was made by Bazina and He [14], who developed a method capable of selectively extracting FFAs from small amounts of fat (100 mg) before their esterification with boron trifluoride (BF_3_). The authors validated the methodology for application to deep-fried fats.

In this study, we validate an effective procedure for derivatization/extraction to accomplish accurate quantitative profiling of EFAs and FFAs from hazelnut fat fraction; moreover, for the first time, quantitative changes in FA profiles from free species as a function of different variables are assessed at a molecular level. The sample-set was designed to cover a wide range of functional variables expected to have an impact on hazelnut FAME composition. In particular, post-harvest drying requires a sensitive method to ascertain the proper inactivation of enzymes and lowering of *a_w_*. The results might help in designing suitable counteractions along the production chain to preserve fat quality over time.

## 2. Materials and Methods

### 2.1. Chemicals and Reference Solutions

Pure standards of *n-*alkanes (from *n-*C7 to *n-*C30) for system evaluation and linear retention index (*I^T^*) determination were purchased from Merck (Milan, Italy).

Process internal standards (P-ISs) heptadecanoic acid (C17:0 (C_17_H_34_O_2_)) and glyceryl tripentadecanoate (C_48_H_92_O_6_) were used for recovery evaluation. Analytical internal standards (A-ISs) normal pentadecane (*n-*C15 (C_15_H_32_)) and normal heptadecane (*n-*C17 (C_17_H_36_)) were used for quality control purposes (i.e., response normalization) and for quantitation through FID response factors (RFs). All ISs were from Sigma Aldrich (Merck, Milan Italy).

The reference mixture of fatty acid methyl esters (FAMEs) for identification and external calibration (Supelco 37 Component FAME Mix), for which detailed composition is provided in Supplementary Table S1, was purchased from Supelco-Merck (Milan, Italy).

Solvents (*n*-hexane and methanol (MeOH)) of LC-MS purity grade were from Merck (Milan, Italy).

Potassium hydroxide (KOH) ACS reagent, ≥ 85%, and sulfuric acid (H_2_SO_4_) ACS reagent, 95.0–98.0%, were from Sigma Aldrich (Merck, Milan, Italy).

### 2.2. Hazelnut Samples

Commercial samples of raw hazelnuts (*Corylus avellana* L.) with the uniform caliber of 13–14 mm, harvested in 2017, were supplied by Soremartec Italia Srl (Alba-Cuneo, Italy). They were from different geographical areas: (a) cultivar Tonda Gentile Trilobata (T) was harvested in Piedmont Italy (IT) and in Georgia (GE), and (b) Georgian cultivar Anakliuri (An) was harvested in Georgia along the Black Sea coast. Hazelnuts were harvested at optimal maturation degree (T0), husked, and dried in-shell under the sun at 38–40 °C (E1) or by artificial dryers at a mild temperature of 18–20 °C (E2) at a final kernel humidity of 6%.

Storage was conducted for 1 year with checkpoints set at 0 months T0, 6 months (T6), and 12 months (T12). During shelf life, two storage conditions were tested: (a) 18 °C with standard atmosphere with 21% O_2_ and 78% N_2_ (18 C) and (b) 5 °C and modified atmosphere with 1% O_2_ and 99% N_2_ (5 V). All samples were kept at 65% of equilibrium relative humidity (ERH).

Raw hazelnuts were manually cut in half for visual quality check, following food technological quality parameters [11], and then ground in a mortar and stored at −18 °C before analysis. Table 1 summarizes hazelnut characteristics and notations adopted across the text.

### 2.3. FAME Quantitative Profiling—Analytical System Configuration and Settings

The analytical system for FAME quantitative profiling consisted of a Thermo Fisher Trace GC Ultra (Milan, Italy) gas chromatograph coupled to a fast flame ionization detector (FID). Sample introduction was accomplished by an auto-sampler model AI 1310 (Thermo Fisher, Milan, Italy) under the following conditions: injector type split/splitless kept at 270 °C, injection mode split, split ratio 1:10, and injection volume 1 μL.

The GC column was a Supelco SLB-IL 76 (tri(tripropylphosphoniumhexanamido)triethylaminebis(trifluoromethanesulfonyl)imide) (30 m × 0.25 mm d*_c_*, 0.20 μm d*_f_*). The carrier gas was helium at a constant flow rate of 1.0 mL/min. The oven temperature program was 60 °C (1 min) to 150 °C (15 min) at 3.0 °C/min and to 240 °C at 3 °C/min (3 min).

Data were acquired and analyzed with Chromaleon version 7.2 SR4 (Thermo Fisher, Milan, Italy).

### 2.4. FAME Identity Confirmation—GC-MS Configuration and Settings

FAME identity was confirmed on a Shimadzu GC2030 system coupled with a triple-quadrupole MS (Shimadzu, Shimadzu Corp, Kyoto, Japan). The MS was operated in single-quadrupole mode in EI mode at 70 eV. The ion source and transfer line temperatures were 200 and 250 °C, respectively.

The GC column was a Supelco SLB-IL 76 (tri(tripropylphosphoniumhexanamido)triethylaminebis(trifluoromethanesulfonyl)imide) (30 m × 0.25 mm d*_c_*, 0.20 μm d*_f_*). The carrier gas was helium at a constant flow rate of 1.0 mL/min. The oven temperature program was 60 °C (1 min) to 150 °C (15 min) at 3.0 °C/min and to 240 °C at 3 °C/min (3 min).

Analyte identity was confirmed by matching EI spectrum with reference spectra in commercial databases and *I^T^* as reported by Dettmer et al. [15].

### 2.5. Fat Extraction

The fat fraction of hazelnuts was extracted with *n-*hexane at ambient temperature and with the aid of ultrasound (US); the extraction exhaustiveness and repeatability were validated in a previous study [16]. In particular, 1.00 g of hazelnut powder was placed in a 20 mL glass vial together with 5.00 mL of *n*-hexane. The extraction took place by means of an ultrasonic bath (Branson 3200; Bransons Ultrasonics, Brookfield, CT, USA) at 40 kHz for 15 min. This procedure was repeated three times, and the *n-*hexane phases were collected and concentrated under a flow of nitrogen. The resulting hazelnut oil was stored at −18 °C before derivatization.

The yield of the *n-*hexane/US extraction protocol was compared with a Soxhlet extraction procedure conducted in agreement with AOAC (association of official analytical chemists) Official Method 948.22 [17] for nuts and nut products. Validation results are discussed in Section 3.1.

### 2.6. Derivatization and Extraction of EFAs and FFAs

The experimental protocol for derivatization and extraction of EFAs and FFAs was based on the protocol of Chau et al. [18]. In particular, an aliquot of the lipid extract obtained by *n*-hexane/US was exactly weighted (0.190–0.220 g) to match with method sensitivity and efficiency. Then, 5 µL of P-ISs (heptadecanoic acid and glyceryl tripentadecanoate at 10 mg/mL) was added.

The first reaction step was aimed at collecting esterified fatty acids (EFAs) and was carried out by transmethylation. Briefly, 2.00 mL of KOH/MeOH 0.4 M solution was added; the resulting mixture was vortex-mixed for 30 s and kept at room temperature for 10 min. Then, a liquid–liquid extraction (LLE) was carried out by adding, in two steps, 2.00 mL of *n-*hexane and vortex-mixing for 30 s each. This process allowed the collection of EFA derivatives in the form of methyl esters into the organic phase. Then, 180 µL over 4.00 mL of total extract was transferred into a 2.0 mL glass vial and spiked with 20 µL of A-IS solution with *n-*C15 and *n-*C17 at 1 mg/mL before GC-FID analysis. The EFA fraction in the final sample corresponded to about 50 mg/mL of total extracted fat.

Fischer esterification was performed on the residual methanolic phase containing FFAs by adding 2.00 mL H_2_SO_4_ 5% to MeOH. The reaction was quantitative at 70 °C for 30 min under constant stirring [18]. FFAs in the form of methyl esters (FAMEs) were then recovered by LLE in two steps by adding 2.00 mL of *n-*hexane and then vortex-mixing for 30 s each. The final organic phase of about 4.00 mL was concentrated under a gentle stream of nitrogen until a final volume of 180 µL. The FFA fraction was then spiked with 20 µL of A-IS solution with *n-*C15 and *n-*C17 at 1 mg/mL before GC-FID analysis. The FFA fraction in the final sample corresponded to about 1 g/mL of total extracted fat.

Supplementary Figure S1 illustrates the derivatization/extraction process.

Recoveries of transmethylation and Fischer esterification were assessed by quantifying C15:0 methyl ester (C_16_H_32_O_2_) in the extracts. Results are reported in Supplementary Table S2 as percentage recovery of C15:0 FAME in the FFA fraction. As clearly shown, the derivatization/extraction efficiency was high with a residual 3.05% of C15:0 FAME recovered in the FFA fraction. The C17:0 FAME process IS adopted for the Fischer esterification reaction was also monitored, but its amount was always below the method limit of detection, confirming a process efficacy close to 100%.

### 2.7. FAME Response Factor Estimation and Recovery Determination

Based on quantitative data reported in the certificate of analysis for reference Supelco 37 Component FAME Mix, FAME FID absolute responses were used to calculate experimental response factors (RFs).

The general formula for RF calculation is reported in (1):

(1) $$RF=\;\frac{A_{ISTD}C_{x}}{A_{X}C_{ISTD}}$$

where *A* indicates the chromatographic area or absolute response of the analyte *x* and *C* is its analytical concentration in µg/mL.

Internal standards for chromatographic repeatability assessment and RF normalization (A-IS) were *n-*C15 and *n-*C17.

Supplementary Table S1 reports analytical concentrations of FAMEs as they are declared by the manufacturer together with their experimental and theoretical predicted FID relative response factors (RRFs) based on combustion enthalpies [19].

Quantitative data for EFAs were expressed as mg of fatty acid/g of oil in agreement with AOAC 963.22 method, while for FFAs, amounts are reported as µg of fatty acid/g of oil. All data are reported as the average values from three replicated injections ± method uncertainty.

### 2.8. Data Analysis and Data Visualization

Statistical data treatment was carried out by XL-STAT (Addinsoft Inc, New York, NY, USA), while heat-map visualization and hierarchical clustering were done by Gene-e Broad Institute [20]. Hierarchical clustering (HC) was based on Pearson correlation distances and was performed after normalization/rescaling of quantitative data on EFA and FFA amounts, expressed as mg/100 g, to a value of 1000.

## 3. Results and Discussion

Hazelnut proximate composition consists of an average of 60% lipids (58.40–64.10 g/100 g), followed by carbohydrates (15.50–17.61 g/100 g), proteins (10.86–16.30 g/100 g), moisture (3.90–5.40 g/100 g), and ash (2.20–2.69 g/100 g). Hazelnut cultivar and the geographical area of harvest greatly impact the bulk composition [21,22,23,24].

Hazelnut lipids are dominated by TAGs combining the six more abundant fatty acids, namely oleic (C18:1 *n*-9), linoleic (C 18:2 *n*-6), palmitic (C 16:0), stearic (C 18:0), linolenic (C 18:2 *n*-3), and arachidic (C 20:0) acids, in different proportions. In particular, TAGs can be found in the hazelnut as glyceryl trioleate (OOO), the most abundant one, followed by 1,2-dioleoyl-3-linoleoyl-glycerol (OOL) and 1-palmitoyl-2,3-dioleoyl-glycerol (POO) [22,23,24,25]. As a consequence, hazelnut kernel is particularly rich in monounsaturated fatty acids (MUFAs) and polyunsaturated fatty acids (PUFAs) and low in saturated fatty acids (SFAs), considering that palmitic and stearic acids represent around 5% and 2% of total fatty acids, respectively [26]. However, the amount of each lipid component is subjected to light quantitative variations based on several factors, i.e., the cultivar, geographical origin, storage conditions, and shelf life, as reported in the literature [25].

Sterols, hydrocarbons, and tocopherols constitute the majority of the unsaponifiable matter [27,28]. Hazelnuts were reported to contain trace amounts of β-, γ-, and δ-tocopherols and high amounts of α-tocopherol (about 30 mg/100 g oil) [29], all of which play an important role in the prevention of lipid oxidation, prolongation of shelf life, and protection of sensory characteristics. The predominant sterol in all hazelnut varieties is β-sitosterol (about 100 mg/100 g oil), followed by campesterol, Δ5-avenasterol, and stigmasterol [30].

For routine analyses, many methods have been developed to appraise the extent and nature of oxidative deterioration of hazelnut lipids. Alasalvar et al. [31] investigated the total fat content; fatty acid composition; and phytosterol, tocopherol, and tocotrienol profiles of Tombul hazelnuts grown in Giresun province. Oliveira et al. [32] focused on the effect of roasting on hazelnut lipids by analyzing raw and roasted hazelnuts and determining their phytosterol and fatty acid (including *trans-*isomers) profiles, TAGs, and tocopherols/tocotrienols distribution.

Furthermore, Ghirardello et al. [4] investigated the effect of different storage conditions on hazelnut quality by measuring moisture, lipid content, total phenolic content, and antioxidant capacity of the kernels and the peroxide value and acidity of the oil. In this perspective, the importance of storage conditions, particularly of vacuum, to stabilize fats during shelf life was highlighted, confirming previous studies by Koyuncu et al. [25].

To the best of the authors’ knowledge, there is a lack of information about the lipolytic stability of hazelnut fats as a function of drying and storage conditions. Therefore, a well-established protocol for differential EFA and FFA quantitative profiling was adapted and validated to enable a more in-depth investigation of the evolution of FFAs.

### 3.1. Extraction Yields and EFAs/FFAs Repartition Ratio

The lipid fraction was extracted in mild conditions to avoid autoxidation processes induced by heating. Therefore, the yield of the *n-*hexane/US extraction protocol was compared with that of a Soxhlet-based procedure conducted in agreement with AOAC Official Method 948.22 [17] for nuts and nut products. The extracted fat was determined by gravimetric analysis, and values were expressed as percentages [33]. Fat extraction yield results are reported in Table 2.

As expected, recoveries were higher with the AOAC method. The continuous extraction process by Soxhlet enables a more efficient extraction from solid particles and lipid stage compartments of nut kernels. However, mild extraction conditions bring several advantages in terms of artifact formation induced by temperature (e.g., *n-*hexane boiling point 69 °C p atm), solvent consumption, and extraction/preparation times. Moreover, the EFA and FFA methyl ester quantitative profiles obtained by the two extraction methods did not reveal meaningful quantitative differences, and the percent error did not exceed 16%, with a median of 4.50% and 5.85% for EFAs and FFAs, respectively. Table 3 reports accuracy data for all quantified FAMEs in the two fractions expressed as percent error values.

### 3.2. Repartition Factor among EFA and FFA Fractions

To validate the specificity and selectivity of the experimental protocol, process internal standards, namely heptadecanoic acid (C17:0) and glyceryl tripentadecanoate, were used to evaluate the recovery by measuring the residual presence of EFAs in the FFA fraction. Of note, for proper calculation of the amounts of glyceryl tripentadecanoate, it has to be considered that the FFA fraction is concentrated to dryness before GC-FID profiling. Moreover, the absence of these two FAMEs was previously assessed on a representative subset of samples.

Results reported as the percentage ratio of the amount of C15:0 FAME among FFAs over the EFAs in all samples (30 samples × 2 extraction batches *n* = 60) and analyzed in triplicate (60 × 3 = 180 analyses) are reported in Supplementary Table S2. The ideal value would be 0%, indicating that the IS C15:0 FAME (derived from glyceryl tripentadecanoate) was quantitatively recovered in the EFA fraction. However, due to its relative solubility in the reaction media, trace amounts could be found in the residual FFA fraction submitted to Fischer esterification.

Results indicated that, on average, the percentage ratio was 3.05 ± 0.85% with a minimum value of 0.87% and a maximum value of 4.87%. Variations were within the method imprecision interval (≈10% relative standard deviation). Quite good selectivity and specificity were achieved, and combined with previous data on extraction yields and profile reproducibility/accuracy, the proposed procedure was applied to the entire batch of samples to study the evolution and quantitative changes in EFA and FFA fractions as functions of different variables.

### 3.3. EFA and FFA Chemical Signatures and Their Informative Potential

The application of the method protocol to all available samples enabled the accurate quantitation of FFAs and EFAs; the data matrix included 30 samples × 2 fractions (*n* = 60) which were measured in triplicate (60 × 3) for a total of 180 analyses. Results are reported as average values of all available replicates, together with absolute uncertainties, in Supplementary Table S3 for EFAs and in Supplementary Table S4 for FFAs.

**Figure 1:**
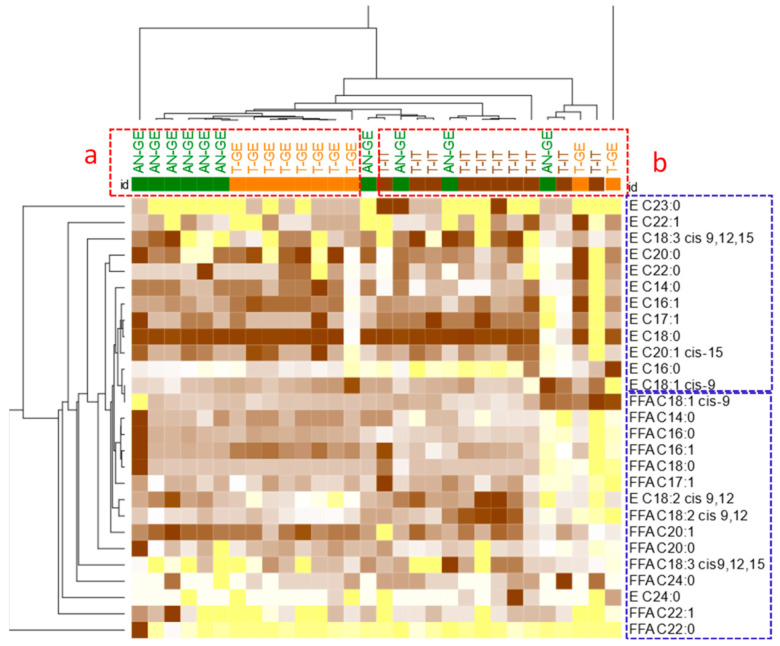
Heat-map visualization of quantitative data referring to EFAs (E) and FFAs (FFA) from analyzed samples (color code: yellow represents minimum value and brown represents maximum value). The hierarchical clustering (HC) was based on Pearson correlation distances and performed after normalization/rescaling of quantitative data, expressed with the same unit as mg/100 g, to 1000. Group “a” clusters together all Georgia samples while Italian harvested samples are grouped in three distinct clusters highlighted in “b”. Simplified sample identifiers are those listed in Table 1.

The simultaneous presence of many different variables with a confounding role interferes with a clear clustering of samples according to cultivar/origin. However, at least for individual components within the EFAs and FFAs, common patterns can be revealed. Figure 1 shows, by heat-map visualization, the normalized quantitative distribution of EFAs and FFAs in all samples. Hierarchical clustering (HC) on all concentrations of FAMEs (from FFAs and EFAs) was based on Pearson correlation distances and was performed after normalization/rescaling of quantitative data, all expressed as mg/100 g, to a value of 1000.

HC results suggest that the two fractions, besides concentration differences, form distinctive patterns (as seen in Figure 1, EFAs in the top-right blue-dotted square and FFAs in the bottom-right blue-dotted square) with common trends within the geographical origin. More marked is, in fact, the signature of FFAs and EFAs within Georgian Anakliuri and Tonda Gentile Trilobata (Figure 1; red dotted square, letter “a”), which is distinct, although not independently clustered, from Tonda Gentile Trilobata from Italy (Figure 1; red dotted square, letter “b”).

When independently explored by supervised statistics, through variable importance in projection (VIP), the highly ranked FFAs with an informative role in discriminating cultivars and harvest region (i.e., T-IT vs. T-GE vs. AN-GE) were myristic (C14:0), palmitoleic (C16:1 *n*-9), linolenic (C18:3 *n*-3), (*Z*)-eicos-11-enoic (C20:1), and behenic (C22:0) acids. On the other hand, within EFAs, those with meaningful variations according to harvest regions were linoleic (C18:2 *n*-6), myristic (C14:0), palmitoleic (C16:1 *n*-9), palmitic (C16:0), and arachidic (C20:0) acids.

Observation of the EFA profiles reveals that they are dominated by oleic acid (C18:1 *n*-9) with an average concentration of 86.2 mg/g, followed by palmitic acid (C16:0) at 6.9 mg/g, linoleic acid (C18:2 *n*-6) at 3.9 mg/g, and stearic acid (C18:0) at 2.22 mg/g. Of them, as discussed above, only linoleic (C18:2 *n*-6) and palmitic (C16:0) acids showed significant variations (*p* < 0.05) between Anakliuri and Tonda Gentile Trilobata cultivars. In particular, linoleic acid (C18:2 *n*-6) was more abundant in Tonda Gentile Trilobata harvested in Italy when compared to the same cultivar harvested in Georgia (4.3 mg/g vs. 3.2 mg/g), while palmitic acid (C16:0) was higher in all Georgian hazelnuts (7.1 mg/g vs. 6.6 mg/g).

Data on FAME profiles are in line with those of Locatelli et al. [34], who studied the acidic signature in raw and roasted Tonda Gentile Trilobata hazelnuts harvested in 2007. These authors reported an average amount of oleic acid (C18:1 *n*-9) of 82 mg/g, followed by almost comparable amounts of linoleic acid (C18:2 *n*-6) at 7.1 mg/g and palmitic acid (C16:0) at 6.5 mg/g. Moreover, Belviso et al. [35], in a study focused on roasting impact on Italian Tonda Gentile Trilobata hazelnuts harvested in 2010 and 2011, reported average amounts of 83.2 (2010) and 80.3 (2011) mg/g for oleic acid (C18:1 *n*-9), 7.0 (2010) and 5.5 (2011) mg/g for palmitic acid (C16:0), 6.4 (2010) and 5.74 (2011) mg/g for linoleic acid (C18:2 n-6), and 2.5 (2010) and 2.2 (2011) mg/g of stearic acid (C18:0).

Considering indicators of lipid quality and oxidative stability [35], the sum (∑) of saturated FAs (∑SCAs), the sum of monounsaturated FAs (∑MUFAs), and the sum of polyunsaturated FAs (∑PUFAs) were computed. Moreover, the (∑MUFA + ∑PUFA)/∑SFA ratio was also considered to evaluate the impact of storage conditions on fat autoxidation. Results are reported in Table 4, where samples are grouped according to cultivar/origin and sub-samples correspond to different post-harvest drying and storage conditions.

Results confirm that the EFA signature was quite stable: ∑SCAs never exceeded 10% relative standard deviation (RSD%). Higher average (∑MUFA + ∑PUFA)/∑SFA ratio values were found in the Tonda Gentile Trilobata samples harvested in Italy (10.2 and 10.6 mg/g for E1 and E2, respectively).

All compositional indicators were comparable to available literature data (e.g., Belviso et al. [35]) and suggested that autoxidation on EFAs was controlled satisfactorily by post-harvest and storage conditions. However, as observed in a previous study focused on the evolution of the volatile fraction as a function of drying and storage [3], secondary products of lipid oxidation might be a more sensitive marker of lipid degradation than quantitative variations in EFA signature.

Oxidative stability is, in fact, correlated with several other variables, e.g., amount and distribution of tocopherols, tocotrienols, and sterols; total phenolic compounds; and presence of transition metals that could catalyze autoxidation of unsaturated fatty acids [36,37].

The next section investigates quantitative (absolute and relative) variations of FFAs as a function of drying and storage time.

### 3.4. Effect of Post-Harvest Treatment on Lipolysis

One of the study’s main objectives was the evaluation of the effect of post-harvest drying and storage conditions on lipolytic activity. Of particular interest was the residual enzymatic activity on TGs, a phenomenon that was expected to have a major influence in hazelnuts with higher residual values of *a_w_*. By observing results reported in Table 4 for the total amount of FFAs, converted in g/100 g of oleic acid (C18:1 *n*-9) equivalents, an increase in acidity during the shelf life can be observed. This parameter, correlated but not equivalent to titratable acidity [14], has no reference values in the available literature [38].

**Figure 2:**
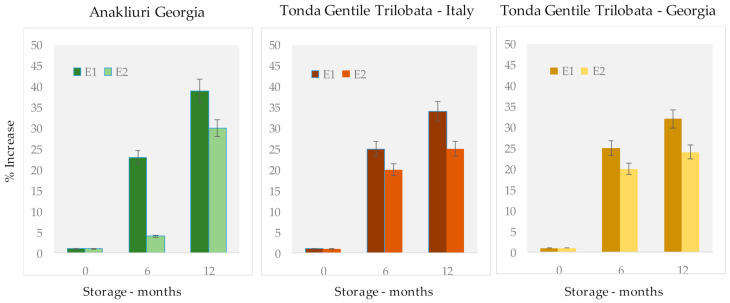
Evolution of total FFA concentration expressed as the percentage increase in oleic acid equivalents (q/100 g) divided/normalized over T0 for Anakliuri and Tonda Gentile Trilobata samples. Dark-color bars correspond to sun drying (E1) and light-color bars correspond to low-temperature drying (E2).

The kinetics of hydrolysis in relation to the post-harvest drying procedure was evaluated by dividing/normalizing the increment in the FFA amount at each time point (T6 and T12) to the value registered at T0. The evolution of ∑FFAs during shelf life for Tonda Gentile Trilobata and Anakliuri hazelnuts is visualized in Figure 2.

Drying conditions (E1 vs. E2) had a major impact on Anakliuri hazelnuts. The oleic acid equivalents increased by 23% after 6 months and achieved a 39% increase after 12 months of storage, independently from temperature (18 or 5 °C) and storage atmosphere composition when sun drying (E1, dark green) was carried out. By applying industrial drying at low temperatures (E2, light green), the FFA increase was limited to 4% in 6 months and reached 30% at the end of the shelf life. Similar trends were observed for Tonda Gentile Trilobata, independently from the harvest region. For this cultivar, the percentage increase in FFAs was less marked, but the effect of drying conditions was consistent with what was observed for Anakliuri hazelnuts.

**Figure 3:**
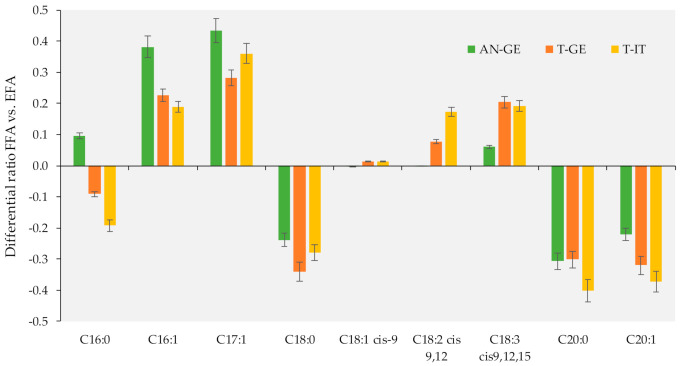
Ratio of FFAs over EFAs, expressed as differential ratio to 1. Fatty acid methyl esters (FAMEs) with an intensity < 0.05% were not considered in the comparison.

Within the FFAs, MUFAs and PUFAs prevailed among the others. Histograms in Figure 3 illustrate the relative ratio of major FFAs for the three sample groups. For each FFA, its average amount (including all time points and conditions) was normalized over the amount of the corresponding species in the EFA fraction. The value was then reported as the difference from 1. With the exception of methyl *cis*-11-eicosenoate (C20:1 *n*-9), all MUFAs and PUFAs in the FFA fraction showed positive variations, leading to the hypothesis that the lipolytic activity of hazelnut lipases is mostly oriented towards the *sn*-2 position of TGs, where MUFAs and PUFAs are mainly present [39].

**Figure 4:**
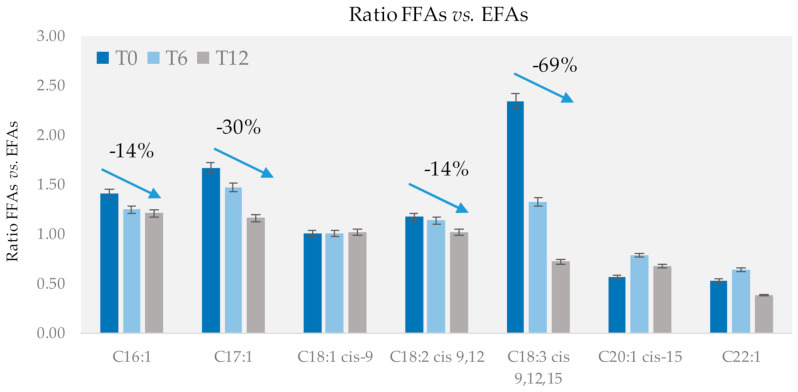
Evolution of the FFAs/EFAs ratio of unsaturated FAs over time.

Moreover, the temporal trend of the FFAs/EFAs ratio indicates that MUFAs and PUFAs are subjected to a progressive decrease during shelf life. The decrease is consistent and more marked for palmitoleic acid (C16:1 *n*-7) and linoleic acid (C18:2, *n*-9,12) with a −14% change in the FFAs/EFAs ratio in 12 months and for *cis*-10-heptadecanoic acid (C17:1 *n*-7) with a −30% change. A more marked decrease was observed for linolenic acid (C18:3 *n*-9,12,15) with a −69% change. Figure 4 shows by histograms the evolution of FFAs/EFAs ratio for selected MUFAs and PUFAs; results are for cumulative data from all cultivars/origins/conditions.

The oxidation rate is highly dependent on the fatty acid structure and, in particular, the number of unsaturations and their positions. Figure 4 shows a combination between the increment in FFAs due to lipase activity and the decrease in the FFAs due to enzymatic or non-enzymatic oxidation. The lower rate observed for C18:2 and the null rate observed for C18:1 are due to the high abundance of these fatty acids in the overall profile; thus, their degradation is partially or entirely compensated by their hydrolytic release. Instead, the high rate of oxidation of the FFAs with three double bonds confirmed the data reported in the literature [12].

## 4. Conclusions

In this study, the fatty acid composition of hazelnuts, both for free and esterified species, was effectively profiled. The derivatization/extraction accompanied by a reliable quantitation by normalized response factors and repartition coefficient corrections enabled quantitative FFA and EFA data to be obtained with great precision and accuracy. Results showed that EFA and FFA signatures are different and follow a clear trend during shelf life. FFAs that showed a meaningful relative difference from EFAs are the unsaturated ones (i.e., palmitoleic acid (C16:1 n-7), *cis*-10-heptadecanoic acid (C17:1 *n*-7), linoleic acid (C18:2, *n*-9,12), and linolenic acid (C18:3 *n*-9,12,15)), suggesting a selective activity of lipases toward the sn-2 position.

The effect of drying conditions on lipolytic stability was confirmed. The incremental trend of the absolute amount of FFAs evidenced a more efficient stabilization provided by industrial dryers operating at lower temperatures when compared to conventional sun drying in-field. During the shelf life, the differential impact of drying led to a 20–40% increase in total FFAs.

The progressive reduction of FFAs (in terms of relative ratio vs. EFAs) during their evolution in the 12 months of storage suggested a concurrent oxidation phenomenon on free forms that, according to reference literature, are readily oxidized. Moreover, the phenomena behind this trend could be multifactorial with additional trigger factors from the enzymatic activity of bacteria and molds.

The methodology proposed in this study could be considered as a further tool for deeply investigating hazelnut chemical composition and stability while supporting industrial investments to preserve kernel quality from post-harvest to late storage.

## Figures and Tables

**Table 1:** Hazelnut samples, characteristics, and notations used in the text.

Cultivar	Geographical Area	Drying	Shelf Life	Storage Condition
Tonda Gentile Trilobata—T	Piedmont, Italy—IT	Conventional—E1 Mild Temperature—E2	T0, T6, T12	5 °C modified atmosphere—5 V 18 °C normal atmosphere—18 C
	Georgia—GE			
Anakliuri—AN	Georgia—GE			

**Table 2:** Crude fat percentage results, with average and relative standard deviation.

Test Portion	AOAC 948.22 Soxhlet Extraction (% Crude Fat)	Mild Extraction *n-*hexane/US (% Crude Fat)
**1**	61.74	54.41
**2**	64.36	51.14
**3**	61.30	55.27
**4**	63.45	56.77
**5**	60.98	52.55
**Average (%)**	**62.37**	**54.03**
**RSD (%)**	**2.35**	**4.11**

**Table 3:** Quantitative data for esterified fatty acids (EFAs) and free fatty acids (FFAs) from a test sample (i.e., blend of different hazelnuts). Percent error was calculated taking the AOAC 984.22 Soxhlet extraction method as benchmark.

Compound	EFAs µg/g			FFAs µg/g		
	Soxhlet	*n*-Hexane/US	Error %	Soxhlet	*n*-Hexane/US	Error %
C14:0	2.72 × 10^1^	2.84 × 10^1^	4.50	7.30 × 10^0^	6.62 × 10^0^	−9.29
C16:0	7.82 × 10^3^	7.54 × 10^3^	−3.53	8.92 × 10^2^	9.32 × 10^2^	4.55
C16:1	3.36 × 10^2^	3.05 × 10^2^	−9.29	5.34 × 10^1^	4.92 × 10^1^	−7.89
C17:1	6.83 × 10^1^	7.62 × 10^1^	11.54	1.84 × 10^1^	1.67 × 10^1^	−9.39
C18:0	3.13 × 10^3^	2.88 × 10^3^	−7.89	2.57 × 10^2^	2.66 × 10^2^	3.55
C18:1 *cis*-9	1.16 × 10^5^	1.05 × 10^5^	−9.39	1.34 × 10^4^	1.38 × 10^4^	3.16
C18:2 *cis* 9,12	1.04 × 10^5^	1.08 × 10^5^	3.55	5.80 × 10^2^	5.67 × 10^2^	−2.31
C18:3 *cis* 9,12,15	2.00 × 10^1^	1.81 × 10^1^	−9.74	7.52 × 10^0^	8.39 × 10^0^	11.56
C20:0	8.83 × 10^1^	9.10 × 10^1^	3.16	1.76 × 10^1^	1.60 × 10^1^	−8.73
C20:1	1.78 × 10^2^	1.74 × 10^2^	−2.31	1.59 × 10^0^	1.44 × 10^0^	−9.29
C22:0	2.44 × 10^1^	2.83 × 10^1^	15.78	2.84 × 10^0^	3.00 × 10^0^	5.52
C22:1	1.79 × 10^0^	1.87 × 10^0^	4.50	9.21 × 10^−3^	1.01 × 10^−2^	10.06
C24:0	2.24 × 10^1^	2.50 × 10^1^	11.54	3.23 × 10^0^	3.41 × 10^0^	5.85

**Table 4:** EFA and FFA cumulative indicators of fat composition. For EFAs, values are reported in mg/g according to Supplementary Table S4 while for ∑FFAs, the calculation refers to the sum of individual FFAs converted in oleic acid equivalents and expressed as g/100 g of fat.

	EFAs mg/g				FFAs g/100 g
Samples ^$^	∑SFA	∑MUFA	∑PUFA	(∑MUFA + ∑PUFA)/∑SFA	∑FFAs Oleic Acid eq.
E1_T0	9.09	86.9	3.99	10.00	1.11
E1_T6_18C	7.93	89.7	2.38	11.61	1.25
E1_T6_5V	9.05	86.4	4.50	10.04	1.63
E1_T12_18C	9.27	86.4	4.30	9.79	1.36
E1_T12_5V	9.54	85.7	4.80	9.49	1.35
**Average**	**8.98**	**87.0**	**3.99**	**10.19**	**1.3**
**RSD%**	**6.84**	**1.8**	**23.74**	**8.10**	**14.4**
E2_T0	9.29	86.8	3.89	9.77	1.28
E2_T6_18C	9.47	85.1	5.42	9.56	1.31
E2_T6_5V	8.77	86.8	4.39	10.41	1.35
E2_T12_18C	9.76	86.0	4.26	9.25	1.92
E2_T12_5V	9.51	85.8	4.71	9.52	1.65
**Average**	**9.36**	**86.1**	**4.54**	**9.70**	**1.5**
**RSD%**	**3.96**	**0.9**	**12.68**	**4.50**	**18.3**
E1_T0	10.12	86.3	3.54	8.88	1.18
E1_T6_18C	9.93	87.0	3.10	9.07	1.59
E1_T6_5V	9.49	87.6	2.95	9.54	1.36
E1_T12_18C	8.53	88.4	3.06	10.73	1.53
E1_T12_5V	9.76	86.7	3.53	9.25	1.54
**Average**	**9.57**	**87.2**	**3.24**	**9.49**	**1.4**
**RSD%**	**6.52**	**0.9**	**8.60**	**7.70**	**11.8**
E2_T0	10.28	86.8	2.91	8.73	1.28
E2_T6_18C	9.88	87.6	2.57	9.12	1.70
E2_T6_5V	9.87	86.7	3.44	9.13	1.39
E2_T12_18C	9.17	87.0	3.84	9.90	1.38
E2_T12_5V	10.27	86.2	3.03	8.70	1.80
**Average**	**9.89**	**86.9**	**3.16**	**9.12**	**1.5**
**RSD%**	**4.54**	**0.6**	**15.58**	**5.34**	**14.8**
E1_T0	8.38	88.2	3.37	10.93	1.01
E1_T6_18C	9.30	88.2	2.47	9.76	1.34
E1_T6_5V	8.48	86.0	5.54	10.79	1.42
E1_T12_18C	11.83	84.4	3.75	7.46	1.63
E1_T12_5V	8.17	86.6	5.25	11.24	1.67
**Average**	**9.23**	**86.7**	**4.08**	**10.04**	**1.4**
**RSD%**	**16.38**	**1.9**	**31.70**	**15.40**	**18.7**
E2_T0	8.74	87.1	4.14	10.44	1.13
E2_T6_18C	8.84	86.3	4.84	10.32	1.26
E2_T6_5V	8.36	87.5	4.18	10.97	1.18
E2_T12_18C	8.90	86.8	4.26	10.24	1.48
E2_T12_5V	8.36	86.0	5.64	10.96	1.46
**Average**	**8.64**	**86.7**	**4.61**	**10.58**	**1.3**
**RSD%**	**3.02**	**0.7**	**13.88**	**3.33**	**12.4**

^$^ Samples acronym are clarified in Table 1.

## Data Availability

The data presented in this study are available in the Supplementary Materials.

## Supplementary Materials

The following are available online at https://www.mdpi.com/2304-8158/10/3/685/s1: Table S1: List of FAME standards and their systematic names, common (trivial) names, abbreviations, reference concentration in the standard mixture, experimental and predicted relative response factors. Table S2: Percentage ratio of the amount of C15:0 FAME among FFAs over the EFAs in all samples (30 samples × 2 extraction batches *n* = 60), analyzed in triplicate (60 × 3 = 180 analyses). Data adapted to normalize analyte amounts. Table S3: EFA amounts expressed as mg/g according to AOAC official method 2003.05. Table S4: FFA amounts expressed as µg/g. Figure S1: Schematic diagram of the derivatization/extraction process.
