# Supplementary material for: Shelf-Life Evolution of the Fatty Acid Fingerprint in High-Quality Hazelnuts (Corylus avellana L.) Harvested in Different Geographical Regions
Source: Foods. 2021 Mar 23;10(3):685. doi: 10.3390/foods10030685 (PMC8004584; doi:10.3390/foods10030685)
Supplement: Supplementary file 1 [file foods-10-00685-s001.pdf]

foods

Supplementary Material

Shelf-life evolution of the fatty acids fingerprint in high-quality hazelnuts (*Corylus avellana* L.) harvested in different geographical regions

Marta Cialiè Rosso[1], Federico Stilo[1], Steven Mascrez[2], Carlo Bicchi[1], Giorgia Purcaro[2*], Chiara Cordero[1*]

[1] Dipartimento di Scienza e Tecnologia del Farmaco, Università degli Studi di Torino, Via Pietro Giuria 9, I-10125 Torino 6707172, Italy; marta.cialierosso@unito.it (M.CR); federico.stilo@unito.it (F.S); carlo.bicchi@unito.it (C.B); chiara.cordero@unito.it (C.C)

[2] AgroBioChem Department, Gembloux Agro-Bio Tech, University of Liège, Passage des Déportés 2, 5030 Gembloux, Belgium; steven.mascrez@uliege.be (S.M); gpurcaro@ulieges.be (G.P.)

* Correspondence: chiara.cordero@unito.it; Tel.: +39 011 6702197 and gpurcaro@ulieges.be; Tel.: +32 (0)81 62 22 20

Abstract: A single paragraph of about 200 words maximum. For research articles, abstracts should give a pertinent overview of the work. We strongly encourage authors to use the following style of structured abstracts, but without headings: (1) Background: Place the question addressed in a broad context and highlight the purpose of the study; (2) Methods: briefly describe the main methods or treatments applied; (3) Results: summarize the article's main findings; (4) Conclusions: indicate the main conclusions or interpretations. The abstract should be an objective representation of the article and it must not contain results that are not presented and substantiated in the main text and should not exaggerate the main conclusions.

Keywords: keyword 1; keyword 2; keyword 3 (List three to ten pertinent keywords specific to the article yet reasonably common within the subject discipline.)

The supplementary material includes:

Supplementary Table 1 - ST1: List of FAMEs standards together with their systematic names, common (trivial) names, abbreviations, reference concentration in the standard mixture, experimental and predicted relative Response Factors.
Supplementary Table 2 – ST2: Results on percentage ratio of the amount of C15:0 FAME among FFA over the EFA in all samples (30 samples x 2 extraction batches n = 60), analyzed in triplicate (60 x 3 = 180 analyses). Data adopted to normalize analytes amounts.
Supplementary Table 3 – ST3: EFAs amounts expressed as mg/g according to AOAC official method 2003.05.
Supplementary Table 4 – ST4: FFAs amounts expressed as µg/g.
Supplementary Figure 1 – SF1: Schematic diagram of the derivatization/extraction process.

foods MDPI

Supplementary Table 1 – ST1: List of FAMEs standards together with their systematic names, common (trivial) names, abbreviations, reference concentration in the standard mixture, experimental and predicted relative Response Factors.

Systematic (IUPAC) name	Common name	Shorthand (ω) name*	Shortened name	Concentration μg/mL Reference mixture	For EFA . n-C15 ref Experimental RF	RRF	For FFA n-C17 ref Experimental RF	RRF
methylbutanoate	methylbutyrate	4:0	C4:0	419	1.497	1.493	1.284	1.526
methylhexanoate	methylcaproate	6:0	C6:0	419	0.941	1.269	0.806	1.297
methyloctanoate	methylcaprylate	8:0	C8:0	417	1.049	1.157	0.899	1.182
methyldecanoate	methyl caprate	10:0	C10:0	426	1.028	1.090	0.881	1.113
methylundecanoate	-	11:0	C11:0	215	1.033	1.065	0.885	1.088
methyldodecanoate	methyllaurate	12:0	C12:0	421	0.985	1.045	0.844	1.067
methyltridecanoate	-	13:0	C13:0	208	0.971	1.028	0.832	1.050
methyltetradecanoate	methylmyristate	10:0	C14:0	423	0.988	1.013	0.847	1.035
methyl*cis*-9-tetradecenoate	methylmyristoleate	14:1 ω-5	C14:1 n-9	211	1.026	1.004	0.880	1.026
methylpentadecanoate	-	15:0	C15:0	209	1.000	1.000	0.857	1.022
methyl*cis*-10-pentadecenoate	-	15:1 ω-5	C15:1 n-10	211	1.099	0.992	0.942	1.014
methylhexadecanoate	methylpalmitate	16:0	C16:0	670	1.103	0.989	0.946	1.010
methyl*cis*-9-hexadecenoate	methylpalmitoleate	16:1 ω-7	C16:1 n-9	210	1.256	0.981	1.077	1.003
methylheptadecanoate	methylmargarate	17:0	C17:0	230	1.167	0.979	1.000	1.000
methyl*cis*-10-heptadecanoate	-	17:1 ω-7	C17:1 n-10	210	1.075	0.972	0.922	0.993
methyloctadecanoate	methylstearate	18:0	C18:0	420	1.159	0.970	0.993	0.991
methyl*trans*-9-octadecenoate	methyl trans-9-elaidate	a	C18:1 n-9	212	1.233	0.964	1.057	0.984
methyl*cis*-9-octadecenoate	methyl oleate	18:1 ω-9	C18:1n-9	422	1.248	0.964	1.070	0.984
methyl *trans*-9-*trans*-12-octadecadienoate	-	a	C18:2 n-9,12t	208	1.184	0.957	1.015	0.978
methyl*cis*-9,*cis*-12-octadecadienoate	methyllinoleate	18:2 ω-6	C18:2 n-9,12	210	1.213	0.957	1.040	0.978
methyl*cis*-6,-9,-12-octadecatrienoate	methyl γ-linolenate	18:3 ω-6	C18:3 n-6,9,12	210	1.132	0.950	0.970	0.971
methyl*cis*-9,-12,-15-octadecatrienoate	methyllinolenate	18:3 ω3-	C18:3 n-9,12,15	209	1.145	0.950	0.981	0.971
methyleicosanoate	methylarachidate	20:0	C20:0	420	1.262	0.955	1.082	0.976
methyl *cis*-11-eicosenoate	methylgondoate	20:1 ω-9	C20:1 n-11	210	1.287	0.949	1.104	0.970
methyl-*cis* -11,-14, eicosadienoate	methyleicosadienoate	20:2 ω-6	C20:2 n-11,14	209	1.350	0.943	1.157	0.964
methylheneicosanoate	-	21:0	C21:0	210	1.442	0.949	1.237	0.969
methyl*cis*-8,-11,-14-eicosatrienoate	methyldihomo- γ -linolenate	20:3 ω-6	C20:3 n-8,11,14	205	1.263	0.937	1.083	0.958
methyl*cis*-5,-8,-11,-14-eicosatetraenoate	methylarachidonate	20:4 ω-6	C20:4 n-5,8,11,14	205	1.280	0.932	1.097	0.952

Systematic (IUPAC) name	Common name	Shorthand (ω) name*	Shortened name	Concentration μg/mL Reference mixture	For EFA Experimental RF	RRF	For FFA Experimental RF	RRF
methyl*cis*-11,14,17-eicosatrienoate	-	20:3 ω-3	C20:3 n-11,14,17	199	1.210	0.937	1.037	0.958
methyldocosanoate	methylbehenate	22:0	C22:0	422	1.374	0.943	1.178	0.963
methylcis-5,-8,-11,-14,-17-eicosapentaenoate	eicosapentaenoate	20:5 ω-3	C20:5 n-5,8,11,14,17	205	1.290	0.926	1.106	0.946
methyl*cis*-13-docosenoate	methylerucate	22:1 ω-9	C22:1 n-13	210	1.389	0.938	1.191	0.958
methyltricosanoate	-	23:0	C23:0	213	0.703	0.938	1.097	0.958
methyltetracosanoate	methyllignocerate	24:0	C24:0	420	1.436	0.933	1.231	0.953
methyl*cis*-15-tetracosenoate	methylnervonate	24:1 ω-9	C24:1 n-15	211	1.454	0.928	1.246	0.948
methyl *cis*-4, -7,-10,-13, -16,-19-docosahexaenoate	methyl DHA	22:6 ω-3	C22:6 n-4,7,10,13,16,19	203	1.403	0.911	1.202	0.930

*Additional methyl group for derivatization as methyl esters to be implied

foods MDPI

Supplementary Table 2 – ST2: Results on percentage ratio of the amount of C15:0 FAME among FFA over the EFA in all samples (30 samples x 2 extraction batches n = 60), analyzed in triplicate (60 x 3 = 180 analyses). Data adopted to normalize analytes amounts.

Sample acronym	Recovery ratio %
An_Ge_E1_T0	2.24
An_Ge_E1_T12_18C	2.24
An_Ge_E1_T12_5V	2.66
An_Ge_E1_T6_18C	2.28
An_Ge_E1_T6_5V	3.27
An_Ge_E2_T0	2.84
An_Ge_E2_T12_18C	3.06
An_Ge_E2_T12_5V	4.87
An_Ge_E2_T6_18C	4.44
An_Ge_E2_T6_5V	2.81
T_Ge_E1_T0	3.60
T_Ge_E1_T12_18C	2.88
T_Ge_E1_T12_5V	2.53
T_Ge_E1_T6_18C	2.89
T_Ge_E1_T6_5V	0.87
T_Ge_E2_T0	3.22
T_Ge_E2_T12_18C	2.66
T_Ge_E2_T12_5V	2.64
T_Ge_E2_T6_18C	4.00
T_Ge_E2_T6_5V	3.52
T_It_E1_T0	2.24
T_It_E1_T12_18C	1.80
T_It_E1_T12_5V	3.85
T_It_E1_T6_18C	3.62
T_It_E1_T6_5V	3.32
T_It_E2_T0	4.21
T_It_E2_T12_18C	3.81
T_It_E2_T12_5V	2.46
T_It_E2_T6_18C	2.58
T_It_E2_T6_5V	3.95
**Average (%)**	**3.05**
**Standard Deviation**	**0.85**

foods

MDPI

Supplementary Table 3 – ST3: EFAs amounts expressed as mg/g according to AOAC official method 2003.05.

Sample Acronym	C14:0	C16:0	C16:1	C17:1	C18:0	C18:1 n-9	C18:2 n-9,12	C18:3 n-9,12,15	C20:0	C20:1 n-15	C22:0	C22:1	C23:0	C24:0
AN_GE_E1_T0	4.05E-02 ± 3.47E-03	6.47E+00 ± 4.92E-01	1.89E-01 ± 1.60E-02	4.65E-02 ± 2.44E-03	2.37E+00 ± 1.51E-01	8.66E+01 ± 5.18E+00	3.93E+00 ± 3.68E-01	6.04E-02 ± 3.53E-03	1.68E-01 ± 9.55E-03	1.06E-01 ± 2.51E-03	2.78E-02 ± 1.82E-03	0.00E+00 ± 0.00E+00	0.00E+00 ± 0.00E+00	1.01E-02 ± 4.61E-04
AN_GE_E1_T12_18C	3.04E-02 ± 2.61E-03	6.79E+00 ± 5.16E-01	2.37E-01 ± 2.00E-02	6.26E-02 ± 3.28E-03	2.35E+00 ± 1.50E-01	8.59E+01 ± 5.14E+00	4.29E+00 ± 4.02E-01	1.13E-02 ± 6.58E-04	6.76E-02 ± 3.85E-03	1.67E-01 ± 3.94E-03	2.90E-02 ± 1.89E-03	6.44E-02 ± 3.84E-03	0.00E+00 ± 0.00E+00	0.00E+00 ± 0.00E+00
AN_GE_E1_T12_5V	3.67E-02 ± 3.14E-03	6.94E+00 ± 5.27E-01	2.38E-01 ± 2.01E-02	5.63E-02 ± 2.95E-03	2.33E+00 ± 1.49E-01	8.52E+01 ± 5.10E+00	4.73E+00 ± 4.43E-01	6.35E-02 ± 3.71E-03	1.56E-01 ± 8.91E-03	1.33E-01 ± 3.15E-03	4.92E-02 ± 3.22E-03	2.69E-02 ± 1.60E-03	1.63E-02 ± 5.56E-03	8.48E-03 ± 3.89E-04
AN_GE_E1_T6_18C	2.13E-02 ± 1.82E-03	6.44E+00 ± 4.89E-01	1.08E-01 ± 9.13E-03	3.09E-02 ± 1.62E-03	1.35E+00 ± 8.60E-02	8.94E+01 ± 5.35E+00	2.35E+00 ± 2.20E-01	3.40E-02 ± 1.99E-03	8.46E-02 ± 4.82E-03	7.32E-02 ± 1.73E-03	1.83E-02 ± 1.20E-03	4.41E-02 ± 2.63E-03	1.15E-02 ± 5.21E-03	1.14E-02 ± 5.22E-04
AN_GE_E1_T6_5V	3.43E-02 ± 2.94E-03	6.52E+00 ± 4.96E-01	2.08E-01 ± 1.76E-02	5.67E-02 ± 2.97E-03	2.36E+00 ± 1.50E-01	8.60E+01 ± 5.15E+00	4.48E+00 ± 4.20E-01	1.50E-02 ± 8.77E-04	7.13E-02 ± 4.06E-03	1.57E-01 ± 3.71E-03	5.93E-02 ± 3.87E-03	3.99E-02 ± 2.38E-03	0.00E+00 ± 0.00E+00	1.29E-02 ± 5.91E-04
AN_GE_E2_T0	3.86E-02 ± 3.31E-03	6.68E+00 ± 5.08E-01	2.26E-01 ± 1.91E-02	5.35E-02 ± 2.80E-03	2.37E+00 ± 1.51E-01	8.64E+01 ± 5.17E+00	3.88E+00 ± 3.63E-01	1.07E-02 ± 6.27E-04	1.62E-01 ± 9.23E-03	1.29E-01 ± 3.06E-03	3.03E-02 ± 1.98E-03	2.92E-02 ± 1.74E-03	0.00E+00 ± 0.00E+00	9.92E-03 ± 4.55E-04
AN_GE_E2_T12_18C	3.81E-02 ± 3.27E-03	7.14E+00 ± 5.42E-01	2.34E-01 ± 1.98E-02	7.03E-02 ± 3.68E-03	2.34E+00 ± 1.49E-01	8.54E+01 ± 5.12E+00	4.20E+00 ± 3.93E-01	6.40E-02 ± 3.74E-03	1.90E-01 ± 1.08E-02	1.81E-01 ± 4.29E-03	3.28E-02 ± 2.14E-03	4.42E-02 ± 2.64E-03	1.01E-02 ± 2.92E-03	9.67E-03 ± 4.44E-04
AN_GE_E2_T12_5V	3.85E-02 ± 3.30E-03	6.93E+00 ± 5.26E-01	2.32E-01 ± 1.97E-02	5.42E-02 ± 2.84E-03	2.34E+00 ± 1.49E-01	8.53E+01 ± 5.11E+00	4.64E+00 ± 4.35E-01	7.01E-02 ± 4.09E-03	1.50E-01 ± 8.52E-03	1.30E-01 ± 3.07E-03	3.03E-02 ± 1.98E-03	5.40E-02 ± 3.22E-03	4.88E-03 ± 1.55E-03	1.48E-02 ± 6.77E-04
AN_GE_E2_T6_18C	4.12E-02 ± 3.53E-03	6.90E+00 ± 5.24E-01	2.51E-01 ± 2.12E-02	5.37E-02 ± 2.81E-03	2.32E+00 ± 1.48E-01	8.47E+01 ± 5.07E+00	5.34E+00 ± 5.00E-01	7.93E-02 ± 4.63E-03	1.63E-01 ± 9.28E-03	1.32E-01 ± 3.12E-03	3.03E-02 ± 1.98E-03	0.00E+00 ± 0.00E+00	0.00E+00 ± 0.00E+00	1.47E-02 ± 6.76E-04
AN_GE_E2_T6_5V	3.12E-02 ± 2.68E-03	6.18E+00 ± 4.70E-01	1.79E-01 ± 1.52E-02	5.60E-02 ± 2.93E-03	2.37E+00 ± 1.51E-01	8.65E+01 ± 5.18E+00	4.32E+00 ± 4.04E-01	7.83E-02 ± 4.57E-03	1.53E-01 ± 8.71E-03	1.12E-01 ± 2.65E-03	2.04E-02 ± 1.33E-03	0.00E+00 ± 0.00E+00	0.00E+00 ± 0.00E+00	1.36E-02 ± 6.24E-04
T_GE_E1_T0	2.76E-02 ± 2.36E-03	7.63E+00 ± 5.80E-01	2.84E-01 ± 2.40E-02	5.34E-02 ± 2.80E-03	2.35E+00 ± 1.50E-01	8.58E+01 ± 5.13E+00	3.53E+00 ± 3.31E-01	1.08E-02 ± 6.29E-04	6.10E-02 ± 3.48E-03	1.84E-01 ± 4.36E-03	2.99E-02 ± 1.96E-03	5.87E-02 ± 3.50E-03	0.00E+00 ± 0.00E+00	1.47E-02 ± 6.75E-04
T_GE_E1_T12_18C	1.68E-02 ± 1.44E-03	6.13E+00 ± 4.66E-01	1.40E-01 ± 1.18E-02	3.88E-02 ± 2.03E-03	1.37E+00 ± 8.73E-02	8.90E+01 ± 5.33E+00	3.01E+00 ± 2.82E-01	5.10E-02 ± 2.98E-03	8.11E-02 ± 4.62E-03	6.99E-02 ± 1.65E-03	2.08E-02 ± 1.36E-03	4.77E-02 ± 2.85E-03	1.25E-02 ± 9.96E-03	3.86E-03 ± 1.77E-04
T_GE_E1_T12_5V	4.45E-02 ± 3.81E-03	7.12E+00 ± 5.41E-01	2.78E-01 ± 2.35E-02	5.48E-02 ± 2.87E-03	2.36E+00 ± 1.51E-01	8.62E+01 ± 5.16E+00	3.48E+00 ± 3.26E-01	5.57E-02 ± 3.25E-03	1.70E-01 ± 9.70E-03	1.31E-01 ± 3.10E-03	5.03E-02 ± 3.29E-03	3.24E-02 ± 1.93E-03	5.51E-03 ± 1.03E-03	8.39E-03 ± 3.85E-04
T_GE_E1_T6_18C	3.80E-02 ± 3.26E-03	7.26E+00 ± 5.52E-01	2.84E-01 ± 2.40E-02	5.41E-02 ± 2.83E-03	2.37E+00 ± 1.51E-01	8.65E+01 ± 5.18E+00	3.04E+00 ± 2.85E-01	5.70E-02 ± 3.33E-03	1.93E-01 ± 1.10E-02	1.27E-01 ± 3.02E-03	5.12E-02 ± 3.35E-03	2.82E-02 ± 1.68E-03	0.00E+00 ± 0.00E+00	1.36E-02 ± 6.26E-04
T_GE_E1_T6_5V	4.52E-02 ± 3.87E-03	6.97E+00 ± 5.30E-01	2.63E-01 ± 2.23E-02	6.58E-02 ± 3.44E-03	2.38E+00 ± 1.52E-01	8.70E+01 ± 5.21E+00	2.94E+00 ± 2.75E-01	1.13E-02 ± 6.60E-04	6.39E-02 ± 3.64E-03	1.99E-01 ± 4.70E-03	6.29E-03 ± 4.11E-04	4.67E-02 ± 2.78E-03	1.24E-02 ± 1.20E-01	7.77E-03 ± 3.56E-04
T_GE_E2_T0	3.60E-02 ± 3.08E-03	7.65E+00 ± 5.81E-01	3.01E-01 ± 2.55E-02	5.29E-02 ± 2.77E-03	2.36E+00 ± 1.50E-01	8.63E+01 ± 5.16E+00	2.88E+00 ± 2.70E-01	2.92E-02 ± 1.71E-03	1.82E-01 ± 1.04E-02	1.86E-01 ± 4.39E-03	3.00E-02 ± 1.96E-03	0.00E+00 ± 0.00E+00	1.18E-02 ± 5.13E-03	8.46E-03 ± 3.88E-04
T_GE_E2_T12_18C	3.76E-02 ± 3.22E-03	6.57E+00 ± 5.00E-01	2.66E-01 ± 2.25E-02	5.84E-02 ± 3.06E-03	2.37E+00 ± 1.51E-01	8.65E+01 ± 5.18E+00	3.79E+00 ± 3.55E-01	4.27E-02 ± 2.49E-03	1.61E-01 ± 9.16E-03	1.31E-01 ± 3.11E-03	2.68E-02 ± 1.75E-03	0.00E+00 ± 0.00E+00	0.00E+00 ± 0.00E+00	0.00E+00 ± 0.00E+00
T_GE_E2_T12_5V	3.06E-02 ± 2.62E-03	1.08E+01 ± 8.17E-01	2.51E-01 ± 2.12E-02	4.75E-02 ± 2.49E-03	2.27E+00 ± 1.44E-01	8.28E+01 ± 4.95E+00	3.47E+00 ± 3.25E-01	3.59E-02 ± 2.09E-03	1.46E-01 ± 8.32E-03	1.13E-01 ± 2.67E-03	3.73E-02 ± 2.44E-03	5.67E-02 ± 3.38E-03	0.00E+00 ± 0.00E+00	2.82E-02 ± 1.29E-03
T_GE_E2_T6_18C	4.02E-02 ± 3.44E-03	7.19E+00 ± 5.47E-01	2.72E-01 ± 2.30E-02	5.00E-02 ± 2.62E-03	2.39E+00 ± 1.52E-01	8.71E+01 ± 5.21E+00	2.52E+00 ± 2.36E-01	4.13E-02 ± 2.41E-03	1.90E-01 ± 1.08E-02	1.26E-01 ± 2.99E-03	3.98E-02 ± 2.60E-03	5.38E-02 ± 3.21E-03	1.49E-02 ± 3.09E-03	1.50E-02 ± 6.89E-04
T_GE_E2_T6_5V	3.89E-02 ± 3.34E-03	7.21E+00 ± 5.48E-01	3.15E-01 ± 2.67E-02	5.69E-02 ± 2.98E-03	2.36E+00 ± 1.50E-01	8.61E+01 ± 5.15E+00	3.39E+00 ± 3.17E-01	4.93E-02 ± 2.88E-03	1.92E-01 ± 1.10E-02	1.25E-01 ± 2.95E-03	5.96E-02 ± 3.90E-03	9.25E-02 ± 5.52E-03	0.00E+00 ± 0.00E+00	1.32E-02 ± 6.05E-04
T_IT_E1_T0	1.77E-02 ± 1.51E-03	6.70E+00 ± 5.09E-01	1.52E-01 ± 1.29E-02	4.44E-02 ± 2.33E-03	1.54E+00 ± 9.84E-02	8.79E+01 ± 5.26E+00	3.33E+00 ± 3.12E-01	3.63E-02 ± 2.12E-03	7.77E-02 ± 4.43E-03	8.42E-02 ± 1.99E-03	2.69E-02 ± 1.76E-03	3.35E-02 ± 2.00E-03	8.06E-03 ± 1.64E-02	7.20E-03 ± 3.30E-04
T_IT_E1_T12_18C	3.94E-02 ± 3.37E-03	9.41E+00 ± 7.15E-01	2.99E-01 ± 2.53E-02	5.89E-02 ± 3.08E-03	2.29E+00 ± 1.46E-01	8.38E+01 ± 5.02E+00	3.74E+00 ± 3.50E-01	1.14E-02 ± 6.64E-04	6.07E-02 ± 3.46E-03	1.70E-01 ± 4.03E-03	7.49E-03 ± 4.89E-04	7.88E-02 ± 4.70E-03	0.00E+00 ± 0.00E+00	1.77E-02 ± 8.14E-04
T_IT_E1_T12_5V	2.48E-02 ± 2.13E-03	5.59E+00 ± 4.25E-01	2.25E-01 ± 1.91E-02	6.19E-02 ± 3.24E-03	2.36E+00 ± 1.50E-01	8.61E+01 ± 5.15E+00	5.16E+00 ± 4.84E-01	8.44E-02 ± 4.92E-03	1.46E-01 ± 8.32E-03	1.74E-01 ± 4.11E-03	2.71E-02 ± 1.77E-03	3.20E-02 ± 1.91E-03	1.12E-02 ± 6.47E-03	8.28E-03 ± 3.80E-04
T_IT_E1_T6_18C	1.38E-02 ± 1.19E-03	8.13E+00 ± 6.18E-01	1.01E-01 ± 8.52E-03	3.15E-02 ± 1.65E-03	1.09E+00 ± 6.97E-02	8.80E+01 ± 5.27E+00	2.44E+00 ± 2.29E-01	3.28E-02 ± 1.92E-03	4.79E-02 ± 2.73E-03	5.08E-02 ± 1.20E-03	8.88E-03 ± 5.80E-04	8.59E-03 ± 5.12E-04	0.00E+00 ± 0.00E+00	0.00E+00 ± 0.00E+00
T_IT_E1_T6_5V	2.40E-02 ± 2.05E-03	5.98E+00 ± 4.54E-01	2.65E-01 ± 2.25E-02	7.10E-02 ± 3.72E-03	2.34E+00 ± 1.49E-01	8.55E+01 ± 5.12E+00	5.52E+00 ± 5.17E-01	1.31E-02 ± 7.65E-04	9.75E-02 ± 5.56E-03	1.30E-01 ± 3.08E-03	2.97E-02 ± 1.94E-03	0.00E+00 ± 0.00E+00	0.00E+00 ± 0.00E+00	1.25E-02 ± 5.72E-04
T_IT_E2_T0	2.34E-02 ± 2.01E-03	6.22E+00 ± 4.73E-01	2.52E-01 ± 2.13E-02	6.29E-02 ± 3.29E-03	2.38E+00 ± 1.51E-01	8.67E+01 ± 5.19E+00	4.13E+00 ± 3.86E-01	1.49E-02 ± 8.72E-04	7.51E-02 ± 4.28E-03	1.44E-01 ± 3.40E-03	2.34E-02 ± 1.53E-03	1.54E-03 ± 9.21E-05	2.06E-02 ± 6.77E-03	0.00E+00 ± 0.00E+00
T_IT_E2_T12_18C	3.19E-02 ± 2.73E-03	6.31E+00 ± 4.79E-01	2.31E-01 ± 1.96E-02	6.75E-02 ± 3.53E-03	2.36E+00 ± 1.51E-01	8.64E+01 ± 5.17E+00	4.25E+00 ± 3.98E-01	1.59E-02 ± 9.25E-04	1.37E-01 ± 7.81E-03	1.48E-01 ± 3.50E-03	4.09E-02 ± 2.68E-03	3.86E-02 ± 2.30E-03	8.04E-03 ± 4.35E-03	1.16E-02 ± 5.34E-04
T_IT_E2_T12_5V	2.77E-02 ± 2.37E-03	5.79E+00 ± 4.40E-01	2.24E-01 ± 1.90E-02	5.66E-02 ± 2.96E-03	2.34E+00 ± 1.49E-01	8.55E+01 ± 5.12E+00	5.57E+00 ± 5.22E-01	6.68E-02 ± 3.90E-03	1.33E-01 ± 7.57E-03	1.22E-01 ± 2.89E-03	4.07E-02 ± 2.66E-03	5.90E-02 ± 3.52E-03	1.73E-02 ± 2.83E-03	1.61E-02 ± 7.39E-04
T_IT_E2_T6_18C	3.04E-02 ± 2.60E-03	6.21E+00 ± 4.72E-01	2.29E-01 ± 1.94E-02	5.94E-02 ± 3.11E-03	2.35E+00 ± 1.50E-01	8.58E+01 ± 5.14E+00	4.76E+00 ± 4.46E-01	7.91E-02 ± 4.62E-03	1.63E-01 ± 9.27E-03	1.66E-01 ± 3.93E-03	3.39E-02 ± 2.21E-03	3.86E-02 ± 2.31E-03	0.00E+00 ± 0.00E+00	5.22E-02 ± 2.40E-03
T_IT_E2_T6_5V	2.40E-02 ± 2.06E-03	5.75E+00 ± 4.37E-01	2.27E-01 ± 1.92E-02	6.01E-02 ± 3.15E-03	2.39E+00 ± 1.52E-01	8.70E+01 ± 5.21E+00	4.11E+00 ± 3.85E-01	6.88E-02 ± 4.01E-03	1.42E-01 ± 8.11E-03	1.48E-01 ± 3.51E-03	3.77E-02 ± 2.46E-03	4.97E-02 ± 2.96E-03	0.00E+00 ± 0.00E+00	2.09E-02 ± 9.57E-04

Supplementary Table 4 – ST4: FFAs amounts expressed as μg/g.

Sample Acronym	C14:0			C16:0			C16:1			C17:1			C18:0			C18:1 n-9			C18:2 n-9,12			C18:3 n-9,12,15			C20:0			C22:0			C22:1			C23:0			C24:0		
AN_GE_E1_T0	6.16	±	0.53	641	±	71.22	30.80	±	1.61	7.98	±	0.51	184	±	11.02	9550	±	894.42	552	±	32.22	9.88	±	0.56	11.60	±	0.27	9.50	±	0.62	3.41	±	0.20	3.50	±	0.00	0.77	±	0.04
AN_GE_E1_T6_18C	3.83	±	0.33	451	±	38.19	17.20	±	0.90	4.68	±	0.30	105	±	6.29	11430	±	789.53	367	±	21.42	7.76	±	0.44	6.39	±	0.15	5.84	±	0.38	2.91	±	0.17	3.54	±	0.01	0.77	±	0.04
AN_GE_E1_T6_5V	6.54	±	0.56	1160	±	98.23	46.80	±	2.45	11.30	±	0.72	269	±	16.10	13900	±	1301.83	748	±	43.66	11.00	±	0.63	19.20	±	0.45	19.70	±	1.29	3.61	±	0.22	0.00	±	0.00	1.78	±	0.08
AN_GE_E1_T12_18C	6.70	±	0.57	1070	±	90.61	40.30	±	2.11	11.60	±	0.74	224	±	13.41	11600	±	1086.42	533	±	31.11	1.48	±	0.08	10.10	±	0.24	15.80	±	1.03	8.86	±	0.53	2.63	±	0.00	1.09	±	0.05
AN_GE_E1_T12_5V	5.62	±	0.48	758	±	64.19	31.90	±	1.67	9.20	±	0.59	170	±	10.18	11830	±	826.99	611	±	35.66	10.20	±	0.58	9.26	±	0.22	10.30	±	0.67	1.25	±	0.07	2.27	±	0.01	4.57	±	0.21
AN_GE_E2_T0	7.32	±	0.63	894	±	75.70	38.00	±	1.99	11.50	±	0.73	213	±	12.75	11000	±	1030.23	518	±	30.23	1.30	±	0.07	0.00	±	0.00	14.30	±	0.93	5.25	±	0.31	0.00	±	0.00	0.24	±	0.01
AN_GE_E2_T6_18C	6.37	±	0.55	952	±	80.62	37.80	±	1.98	11.50	±	0.73	215	±	12.87	11200	±	1048.96	509	±	29.71	4.49	±	0.26	14.30	±	0.34	18.80	±	1.23	15.00	±	0.90	7.41	±	0.00	4.79	±	0.22
AN_GE_E2_T6_5V	7.10	±	0.61	916	±	77.57	31.70	±	1.66	14.90	±	0.95	219	±	13.11	11400	±	1067.69	757	±	44.18	17.50	±	1.00	12.90	±	0.31	11.20	±	0.73	0.78	±	0.05	2.26	±	0.00	2.86	±	0.13
AN_GE_E2_T12_18C	14.80	±	1.27	2280	±	193.07	86.70	±	4.54	18.10	±	1.15	540	±	32.33	15000	±	1098.12	813	±	47.45	6.75	±	0.38	41.70	±	0.99	19.60	±	1.28	85.80	±	5.12	7.49	±	0.00	2.24	±	0.10
AN_GE_E2_T12_5V	7.78	±	0.67	1230	±	104.16	47.80	±	2.50	8.61	±	0.55	275	±	16.46	14200	±	1329.93	527	±	30.76	2.44	±	0.14	7.93	±	0.19	20.00	±	1.31	7.20	±	0.43	4.47	±	0.00	2.18	±	0.10
T_GE_E1_T0	6.60	±	0.57	901	±	76.30	45.00	±	2.36	11.60	±	0.74	193	±	11.55	10000	±	936.57	477	±	27.84	10.20	±	0.58	13.70	±	0.32	8.40	±	0.55	2.09	±	0.12	1.00	±	0.00	1.26	±	0.06
T_GE_E1_T6_18C	8.56	±	0.73	1180	±	99.92	55.10	±	2.89	12.50	±	0.80	262	±	15.68	13600	±	1273.73	573	±	33.44	10.80	±	0.62	20.10	±	0.48	16.70	±	1.09	6.75	±	0.40	2.97	±	0.00	2.73	±	0.13
T_GE_E1_T6_5V	7.95	±	0.68	1070	±	90.61	45.20	±	2.37	12.00	±	0.76	223	±	13.35	11600	±	1086.42	448	±	26.15	1.46	±	0.08	12.20	±	0.29	16.90	±	1.10	3.29	±	0.20	0.00	±	0.12	2.86	±	0.13
T_GE_E1_T12_18C	8.73	±	0.75	1220	±	103.31	57.90	±	3.03	10.00	±	0.64	249	±	14.91	12900	±	1208.17	691	±	40.33	4.35	±	0.25	17.30	±	0.41	16.60	±	1.09	2.93	±	0.17	0.72	±	0.01	2.80	±	0.13
T_GE_E1_T12_5V	7.09	±	0.61	1090	±	92.30	53.50	±	2.80	13.30	±	0.85	255	±	15.27	13200	±	1236.27	583	±	34.03	7.35	±	0.42	17.20	±	0.41	17.00	±	1.11	8.30	±	0.50	3.78	±	0.00	3.95	±	0.18
T_GE_E2_T0	7.23	±	0.62	971	±	82.22	49.60	±	2.60	8.85	±	0.56	211	±	12.63	11000	±	1030.23	422	±	24.63	8.67	±	0.49	14.50	±	0.34	12.10	±	0.79	2.60	±	0.16	0.00	±	0.01	2.29	±	0.11
T_GE_E2_T6_18C	9.75	±	0.84	1260	±	106.70	56.20	±	2.94	12.10	±	0.56	282	±	16.88	14600	±	1367.39	555	±	32.39	11.30	±	0.64	24.10	±	0.57	18.30	±	1.20	5.95	±	0.36	5.55	±	0.00	2.83	±	0.13
T_GE_E2_T6_5V	5.74	±	0.49	701	±	59.36	36.60	±	1.92	7.71	±	0.49	145	±	8.68	12490	±	1169.77	376	±	21.95	6.66	±	0.38	10.30	±	0.24	9.52	±	0.62	3.14	±	0.19	0.00	±	0.00	1.32	±	0.06
T_GE_E2_T12_18C	6.76	±	0.58	936	±	79.26	50.40	±	2.64	7.51	±	0.48	229	±	13.71	11900	±	1114.52	563	±	32.86	1.50	±	0.09	10.10	±	0.24	15.00	±	0.98	2.29	±	0.14	0.00	±	0.00	1.10	±	0.05
T_GE_E2_T12_5V	4.49	±	0.38	576	±	48.78	26.90	±	1.41	4.41	±	0.28	131	±	7.84	16770	±	1570.63	367	±	21.42	5.46	±	0.31	7.69	±	0.18	8.10	±	0.53	2.85	±	0.17	2.50	±	0.00	1.00	±	0.05
T_IT_E1_T0	2.50	±	0.21	334	±	28.28	18.60	±	0.97	4.30	±	0.27	100	±	5.99	9200	±	955.30	389	±	22.70	5.08	±	0.29	4.29	±	0.10	9.39	±	0.61	2.63	±	0.16	2.47	±	0.02	5.01	±	0.23
T_IT_E1_T6_18C	5.97	±	0.51	856	±	72.49	33.60	±	1.76	7.81	±	0.50	224	±	13.41	11600	±	1086.42	508	±	29.65	8.15	±	0.46	9.42	±	0.22	8.53	±	0.56	5.43	±	0.32	4.26	±	0.00	4.07	±	0.19
T_IT_E1_T6_5V	4.53	±	0.39	868	±	73.50	38.40	±	2.01	13.40	±	0.85	238	±	14.25	12300	±	1151.98	607	±	35.43	1.82	±	0.10	8.67	±	0.21	17.00	±	1.11	10.90	±	0.65	3.47	±	0.01	3.59	±	0.16
T_IT_E1_T12_18C	2.45	±	0.21	332	±	28.11	16.80	±	0.88	4.21	±	0.27	104	±	6.23	15410	±	1443.25	406	±	23.70	6.34	±	0.36	5.15	±	0.12	6.44	±	0.42	2.58	±	0.15	0.00	±	0.00	6.86	±	0.31
T_IT_E1_T12_5V	4.87	±	0.42	966	±	81.80	52.10	±	2.73	10.40	±	0.66	273	±	16.34	14100	±	1320.56	1150	±	67.12	1.59	±	0.09	0.00	±	0.00	13.00	±	0.85	1.88	±	0.11	0.00	±	0.00	2.05	±	0.09
T_IT_E2_T0	6.62	±	0.57	532	±	78.92	49.20	±	2.58	16.70	±	1.06	266	±	15.92	9800	±	1292.47	567	±	33.09	8.39	±	0.48	16.00	±	0.38	1.44	±	0.09	3.00	±	0.18	0.01	±	0.01	3.41	±	0.16
T_IT_E2_T6_18C	5.32	±	0.46	759	±	64.27	35.70	±	1.87	12.30	±	0.78	204	±	12.21	10600	±	992.76	811	±	47.33	12.30	±	0.70	11.70	±	0.28	13.50	±	0.88	8.66	±	0.52	3.95	±	0.00	3.35	±	0.15
T_IT_E2_T6_5V	4.65	±	0.40	738	±	62.49	37.10	±	1.94	10.90	±	0.69	191	±	11.43	9880	±	925.33	772	±	45.06	8.80	±	0.50	10.40	±	0.25	13.30	±	0.87	3.28	±	0.20	1.79	±	0.00	0.92	±	0.04
T_IT_E2_T12_18C	7.39	±	0.63	916	±	77.57	28.90	±	1.51	12.90	±	0.82	249	±	14.91	12900	±	1208.17	573	±	33.44	1.82	±	0.10	10.40	±	0.25	13.60	±	0.89	6.11	±	0.36	5.03	±	0.00	3.53	±	0.16
T_IT_E2_T12_5V	5.84	±	0.50	902	±	76.38	44.00	±	2.30	12.50	±	0.80	236	±	14.13	12200	±	1142.61	1030	±	60.12	13.90	±	0.79	11.30	±	0.27	17.30	±	1.13	2.27	±	0.14	3.36	±	0.00	2.71	±	0.12

Supplementary Figure 1 – SF1: Schematic diagram of the derivatization/extraction process.

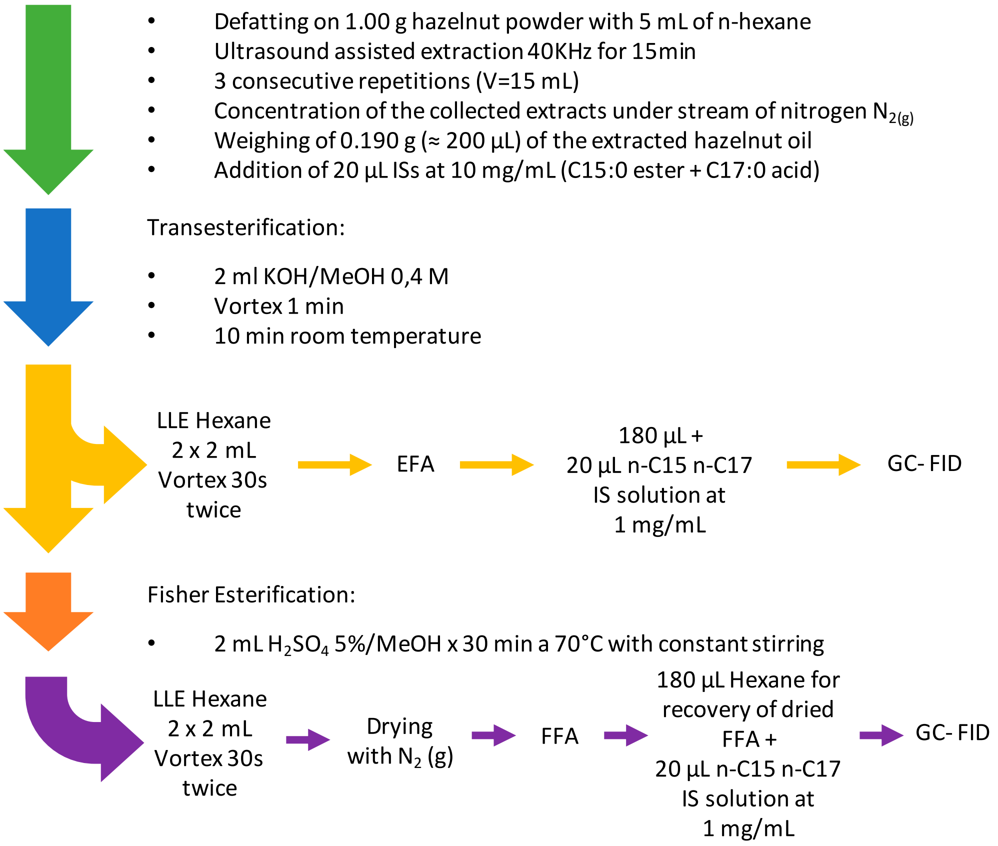

Author Contributions: Conceptualization, Carlo Bicchi, Giorgia Purcaro and Chiara Cordero; Data curation, Marta Cialiè Rosso, Federico Stilo, Giorgia Purcaro and Chiara Cordero; Formal analysis, Marta Cialiè Rosso and Steven Mascrez; Funding acquisition, Carlo Bicchi; Investigation, Chiara Cordero; Methodology, Giorgia Purcaro; Supervision, Chiara Cordero; Writing – original draft, Federico Stilo, Giorgia Purcaro and Chiara Cordero; Writing – review & editing, Carlo Bicchi, Giorgia Purcaro and Chiara Cordero.

Funding: This research was funded by Soremartec Italia Srl, Project: Shelling nuts: a comprehensive investigation of hazelnuts flavor, taste and related health topics.

Data Availability Statement: The data presented in this study are available in supplementary material here.

Conflicts of Interest: The authors declare no conflicts of interest
